# A Wake-Up Call: Recognizing and Reimaging Responses to Children's Mental Health and Protection Needs as an Integral Part of the COVID-19 Pandemic

**DOI:** 10.3389/fpsyt.2022.841515

**Published:** 2022-02-10

**Authors:** Mireia Solerdelcoll

**Affiliations:** ^1^Department of Child and Adolescent Psychiatry, Institute of Psychiatry, Psychology and Neuroscience, King's College London, London, United Kingdom; ^2^Department of Medicine, University of Barcelona, Barcelona, Spain

**Keywords:** COVID-19, child development, child protection, mental health, culture, resilience, vulnerable population

## Introduction

The coronavirus disease 2019 (COVID-19) pandemic is an unprecedented worldwide crisis that presents unprecedented risks to the safety, wellbeing and development of the world's children. The pandemic-related economic breakdown and psychosocial impact have increased the existing child psychosocial needs and highlighted the weaknesses of mental health systems globally (1). Moreover, the harmful effects of COVID-19 and related control measures are not distributed equally, and the most damaging and longest-lasting consequences are likely to impact those who are already most disadvantaged or vulnerable (2). The pandemic is also exposing the fact that the harmful effects are not equally distributed and particular vulnerable and already disadvantaged groups, such as children from low-income and middle-income countries (LMICs), those with neuropsychiatric conditions or disabilities, and those in vulnerable social circumstances (for instance, refugee or migrant children, and children living in alternative care or overcrowded settings), might be at even greater risk of poor educational, social and mental health outcomes because pre-existing failures in human rights protection are worsened (3, 4).

If the pandemic has taught us anything, it is that mental health is profoundly affected by the world around us. Socio-economic and cultural factors and humanitarian crises like the COVID-19 pandemic can lead to severe and lasting psychosocial distress (5). Children are subject to multiple influences throughout their lives, and mental health is also a reflection of their circumstances, personal experiences, and cultural context (6). This opinion article provides an overview of the foundational role of culture and environmental contexts on children's mental health and wellbeing under the COVID-19 circumstance and discusses recommendations and considerations on how culturally sensitive approaches can promote and protect mental health and care for the most vulnerable youth in these challenging times.

## Spheres of Influence on Children's Mental Health and Wellbeing

Culture and contexts shape how mental disorders are experienced, understood and addressed. For children and young people understanding mental health means recognizing that is entwined with societal and family values, cultural standards, social expectations, and developmental capacities (7). For example, perceptions of acceptable behavior, appropriate social functioning, expectations of happiness and personal growth, and experiences of adversity are understood differently in different cultural contexts, affecting understanding of mental health and wellbeing. As a result, the successful implementation of mental health policies and programmes need to take into consideration familiar, cultural, social, political, and environmental contexts. When these different perspectives and cultural contexts inform responses to mental health, they can lead to prioritizing interventions that are more beneficial, acceptable and that build on the strengths of those societies (8).

Over time and as the pandemic unfolds, children are subject to multiple influences that lead to positive and negative trajectories. These differences often stem from cultural stereotypes and norms, powerful social determinants of mental health, but are also linked to family and community behaviors and expectations (9).

Recognizing a socio-ecological framework, multiple factors shape the mental health of children and youth (10). It is molded by the worlds of parents and caregivers, communities and schools, and large-scale social determinants such as poverty, conflict, and pandemics (11). At critical moments of child development, these experiences and environments can contribute to pre-existing vulnerabilities and harm mental health and wellbeing, but they also offer unique opportunities to promote and protect mental health and resilience (7, 12). A recent UNICEF report synthesized six life domains that have the greatest impact on children's development: physical and mental health and wellbeing; economy and equality; learning and human capital formation; violence and conflict; family relationships and social networks (9).

## The Role of Parenting in Addressing Mental Health and Psychosocial Aspects

The role of parenting is foundational to children's mental health (13). Positive parenting skills become even more important when schools are closed, social services are disrupted, and children are confined at home. Positive parenting can foster adolescents' capacity for resilience in the face of adversity and have long-lasting effects on health and education. Across diverse cultural contexts, warm relationships between caregivers and children can lead to positive outcomes, including higher self-esteem, reduced stress, better mental health and fewer psychological and behavior problems (14, 15). However, parents' psychological distress reduces their parenting skills and leads to impaired parent-child interactions (16). A key approach to laying the foundation for youth mental health requires providing care for caregivers and encouraging engaged parenting. To achieve good mental wellbeing as part of COVID-19 response efforts, we must ensure that the family's basic needs are met and that the rights of children are fully promoted and protected. Supporting parents and caregivers should be addressed through collaborative networks between key actors such as governments and local authorities, intergovernmental organizations, community-based and civil society actors, and healthcare professionals, by the implementation of social protection measures that include financial and nutritional support, family-friendly policies, and parent training and counseling programs. When social inequalities remain unaddressed, mental health interventions are less effective. Thus, inequality is an important determinant of mental health, and it is worsening as a result of the pandemic. Mental health and psychosocial response require recognition of the syndemic co-occurrence and interaction of health burdens and sociocultural context (17). Family environments marked by poverty or limited resources will bear the full brunt of COVID-19 and associated containment measures (3). High-stress home environments increase the likelihood of family conflicts, as well as emotional and behavioral problems, domestic abuse and violence. Therefore, the effectiveness of public health strategies and programmes in global mental health should strategically address the social determinants (6). Enabling families to cope with this situation will require reducing stressors and economic instability and increasing parenting capabilities, along with mental health support. An effective COVID-19 response promotes responsive caregiving and nurturing connections, ensures mental health care for caregivers, and provides parents training to respond to children's mental health challenges. Mental health programmes must prioritize caregivers by providing support to manage stress and conflict and enhancing coping strategies, prioritizing targeted support for families and children at particular risks, such as those facing violence and groups with previous vulnerabilities (e.g., mental and substance use disorders) (18).

## Education Environments

Information, education, and online materials on mental health and psychosocial wellbeing have been produced by many institutions and organizations to provide immediate help and coping strategies for managing infection containment-related psychological stress during the COVID-19 pandemic and beyond (19). Despite the window of opportunity presented by the use of digital technology for various mental health care tasks, caution is needed as barriers to access and use of different telehealth resources may increase existing disparities in access to mental health care. Two-thirds of the world's school-age children have no internet connection at home and in the context of COVID-19 and prolonged school closures, it can lead to isolation, loss of learning, and perpetuate inequalities that already divide youth from the poorest households, rural areas, and LMICs from their peers. Aside from the important academic benefits of schooling, schools have an essential role in shaping the mental wellbeing of young people by providing a structured and supervised space for socioemotional development, friendship and social support networks, protection from risk-taking behaviors, and often represent a key access point for food, which negatively affects mental health (1).

Schools provide a critical platform to promote and protect children's and young people's mental health and reach children in need of care, those who might otherwise not have access to mental health services. In countries at all income levels, especially in LMICs, evidence shows that school-based interventions that focus on developing social, emotional, problem-solving and coping skills are linked to mental health benefits. As a result, learning environments are critical platforms where cost-effective and culturally acceptable interventions can foster inclusion and promote and protect mental health (20, 21).

We suggest that an inter-agency collaboration between child protection actors, education, and mental health professionals should be adopted in the design and development of programs for preventing, identifying, and delivering psychosocial support to children and adolescents and their caregivers (22).

## Experiences of Stigma and Discrimination

Despite growing media and community awareness of the importance of mental health and the establishment of children's mental health as a core priority of governments and other stakeholders, it is still widely stigmatized and misunderstood. The influences of stigma on mental health are complex and can work bi-directionally, instigating and exacerbating mental health conditions (23). Additionally, stigma can intersect and combine with other forms of discrimination, such as gender, race, socioeconomic status, sexual orientation, or disability, to lead to poor mental health and hamper efforts to promote mental health and protect vulnerable children and youth. Mental health is widely stigmatized and influenced by laws, policies, the media, attitudes, and cultural norms that perpetuate stereotypes and limit opportunities for children and youth to grow, learn, and prosper (24, 25).

Against a backdrop of rising awareness of mental health issues, there is now an opportunity to promote good mental health for every child, protect vulnerable children, and care for children facing the greatest challenges. Making that happen will require not only investment in mental health across multiple sectors, including primary health care, education, and social protection sectors, but also requires societies to break the silence surrounding mental health, by addressing stigma, promoting understanding and a positive state of wellbeing for children and their caregivers (6).

Racial discrimination devalues, disempowers and, whether felt directly or indirectly, significantly harms children's mental health and wellbeing. Research suggests that the effect of racism on mental health is profound, complex, entrenched and pervasive (26, 27). Racism is a social determinant of health that has a profound impact on health status and affects mental health in multiple ways: by disparities in educational access and outcomes; increased and prolonged levels of exposure to stress; the internalization of negative stereotypes related to race and damaging self-esteem; disadvantage and discrimination as a factor limiting the access to health care, and can also impact the diagnosis of mental health conditions. Perceived racial discrimination is associated with increased symptoms of depression, anxiety, post-traumatic stress disorder, poor sleep, reduced self-regulation, and increased rates of risky behaviors, substance use or delinquency. Additionally, experiences of racism can result in a downstream effect of racial inequality, such as intergenerational and historical trauma (27). Thus, recognizing the cultural background as an integral part of the person and addressing the roots of discrimination is essential to safeguard mental health. A core commitment to a culturally sensitive approach to mental health care for young people and adjusted to their particular needs and challenges must be a guiding principle. Recent mental health guidelines call on practitioners to raise their awareness of discrimination; conduct culturally aware assessments; establish an individualized and culturally appropriate treatment approach; and provide multidisciplinary assistance (28).

## Resilience

Human resilience depends on a combination of multiple biological, psychological, social, and ecological systems interacting as a combination of co-occurring and co-dependent elements in ways that help individuals to regain, sustain, or improve their mental wellbeing in contexts of adversity (29). In the context of exposure to adversity, Ungar argued that resilience is both the capacity of individuals to navigate their way to the psychological, social, cultural, and physical resources that sustain their wellbeing, and their capacity individually and collectively to negotiate for these resources to be provided and experienced in culturally meaningful ways (30). Resilience requires the ability to navigate seven tensions: access to material resources; relationships; power and control; social justice and cohesion; and being especially relevant for this opinion article: a personal and collective sense of identity, purpose and belonging; and cultural adherence and cohesion to local, global or cultural practices, beliefs, including religious beliefs, and values (31). Fostering resilience in children and youth requires recognizing the unique qualities in diverse cultural forms with specific values and concepts of mental health, needs, and resources (32).

While the measures adopted by public health authorities to prevent and control the spread of COVID-19 were necessary to reduce the disease burden, accumulating evidence suggests that pandemic-related restrictions have resulted in an increase in known risk factors for mental health problems and have contributed to the risk of deepening social, educational, and health inequalities, exacerbating pre-existing vulnerabilities for marginalized and disadvantaged children and families (33). Overall, the data published to date suggest that while some children demonstrated resilience, a significant cohort of children and adolescents appear to be experiencing psychological distress related to the COVID-19 crisis (34, 35). As the pandemic has progressed, referrals to child mental health services have increased, reaching record demands (36).

Studies of resilience show that regulatory capacities and changes to cognitions are unsustainable unless other co-occurring social and physical resources, such as family, housing, and natural environment are adequate and facilitated (37). The promotive and protective factors and processes enable resilience when they express sensitivity to contextual and cultural dynamics. Thus, heightened awareness of how resilience interventions can meaningfully respond to the COVID-19 pandemic has the potential to enable mental health practitioners to better support positive mental health outcomes in children. To this end, mental health professionals need to work in multidisciplinary teams to promote interventions that promote resilience tailored to the cultural and contextual norms of different populations who can facilitate access to protective socioecological supports while treating disorders (38).

## Conclusions and Future Directions

To promote good mental health for every child, protect vulnerable children and care for children facing the greatest challenges, we outline a framework on the foundation of three core principles: commitment to strengthening leadership and partnerships, backed by investment in supporting mental health; Communication to break down stigmas around mental health, opening conversations and improving mental health literacy; and Action to strengthen the capacity of health, education, social protection and other workforces, to better support families, schools and communities, and to improve data, research and evidence (6).

The impact of the COVID-19 pandemic on children's mental health has raised serious concerns and has generated an enormous amount of speculation, media coverage and academic studies. So far, no event has aroused so much interest in the global media about the state of mental health as now. Part of the media coverage of COVID-19 has focused on disseminating information and raising awareness about the effects of the pandemic on mental health, however, a framework for action is needed (39).

This opinion article aimed to deepen knowledge and raise awareness of the key cultural and contextual factors affecting children and advocate for responses that improve children's lives in the current context. Hence, after governments, academics, and other stakeholders have shown a public commitment and broad communication to better mental health, we crucially conclude by calling governments, health institutions, and non-governmental organizations to commit to act in key areas and prioritize the needs of children and young people in any implementation of COVID-19 responses. If children's needs are not appropriately addressed, the mental health consequences for a generation of children could far exceed the immediate health and economic impact of the COVID-19 pandemic, leaving long-term social and economic consequences (22).

## Author's Note

The content of this manuscript has been presented in part at the UNICEF report on The State of the World's Children 2021 (6).

## Author Contributions

The author confirms being the sole contributor of this work and has approved it for publication.

## Funding

MS receives grant support from the Alicia Koplowitz Foundation.

## Conflict of Interest

The author declares that the research was conducted in the absence of any commercial or financial relationships that could be construed as a potential conflict of interest.

## Publisher's Note

All claims expressed in this article are solely those of the authors and do not necessarily represent those of their affiliated organizations, or those of the publisher, the editors and the reviewers. Any product that may be evaluated in this article, or claim that may be made by its manufacturer, is not guaranteed or endorsed by the publisher.

## Abbreviations

COVID-19: coronavirus disease 2019
LMICs: low-income and middle-income countries.
